# Hepatitis B Virus-Encoded X Protein Downregulates EGFR Expression via Inducing MicroRNA-7 in Hepatocellular Carcinoma Cells

**DOI:** 10.1155/2013/682380

**Published:** 2013-06-11

**Authors:** Yun-Ju Chen, Pei-Hsuan Chien, Wen-Shu Chen, Yu-Fong Chien, Ya-Ying Hsu, Li-Yun Wang, Jhen-Yu Chen, Chih-Wen Lin, Tzung-Chi Huang, Yung-Luen Yu, Wei-Chien Huang

**Affiliations:** ^1^Department of Biological Science and Technology, I-Shou University, Kaohsiung 824, Taiwan; ^2^Department of Medical Research, E-Da Hospital, Kaohsiung 824, Taiwan; ^3^Center for Molecular Medicine, China Medical University Hospital, Taichung 404, Taiwan; ^4^Graduate Institute of Cancer Biology, China Medical University, Taichung 404, Taiwan; ^5^The School of Chinese Medicine for Post-Baccalaureate, I-Shou University, Kaohsiung 824, Taiwan; ^6^Division of Gastroenterology and Hepatology, Department of Medicine, E-Da Hospital, Kaohsiung 824, Taiwan; ^7^Department of Biomedical Imaging and Radiological Science, China Medical University, Taichung 404, Taiwan; ^8^The Ph.D. Program for Cancer Biology and Drug Discovery, China Medical University, Taichung 404, Taiwan; ^9^Department of Biotechnology, Asia University, Taichung 413, Taiwan

## Abstract

Hepatitis B virus (HBV) infection accounts for over a half of cases of hepatocellular carcinoma (HCC), the most frequent malignant tumor of the liver. HBV-encoded X (HBx) plays critical roles in HBV-associated hepatocarcinogenesis. However, it is unclear whether and how HBx regulates the expression of epidermal growth factor receptor (EGFR), an important gene for cell growth. Therefore, the study aimed to investigate the association between HBx and EGFR expression. In this study, we found that HBx upregulates miR-7 expression to target 3′UTR of EGFR mRNA, which in turn results in the reduction of EGFR protein expression in HCC cells. HBx-mediated EGFR suppression renders HCC cells a slow-growth behavior. Deprivation of HBx or miR-7 expression or restoration of EGFR expression can increase the growth rate of HCC cells. Our data showed the miR-7-dependent EGFR suppression by HBx, supporting an inhibitory role of HBx in the cell growth of HCC. These findings not only identify miR-7 as a novel regulatory target of HBx, but also suggest HBx-miR-7-EGFR as a critical signaling in controlling the growth rate of HCC cells.

## 1. Introduction

 Hepatocellular carcinoma (HCC), the third leading cause of cancer-associated death worldwide, is a heterogeneous and complex disease [1]. Chronic infections of hepatitis virus, such as hepatitis B virus (HBV) and hepatitis C virus (HCV), are known to contribute to the tumorigenesis in most of HCC [2]. Particularly, HBV infection-associated HCC accounts for over a half of HCC cases and is endemically observed in Asia and Africa [3, 4]. HBV-associated hepatocyte transformation is attributed to inflammatory responses, destruction and regeneration of hepatocytes, and pleiotropic activities of HBV-encoded proteins [5]. When HBV-infected insults are destroyed, hepatocyte regeneration is activated for the replacement of damaged or destroyed hepatocytes by replication of mature hepatocytes [6]. Similar to wound healing, deposition of extracellular matrix components occurrs during liver regeneration and thereby causes liver fibrosis and cirrhosis [7]. In the potentially mutagenic environment caused by continual inflammation, repeated proliferation of hepatocytes and constant liver regeneration may eventually be selected for transformed hepatocytes and could link HBV infections to the development of HCC [6].

In addition to HBV-initiated immune and inflammatory responses, HBV-encoded proteins *per se* may also regulate proliferation and regeneration of hepatocytes by altering multiple cellular signaling transduction pathways [8]. The HBV genome contains four overlapping open reading frames (ORFs), which encode pre-S1/pre-S2/S, viral polymerase, HBV X protein (HBx), and pre-C/C, respectively. Among them, the HBx protein is the smallest one with 154 amino acids and is thought to make the most significant contribution to the development of HBV-associated HCC [9, 10]. However, the roles of HBx in proliferation, apoptosis, and liver regeneration remain controversial. Results from two studies using transgenic HBx mouse models reveal its oncogenic function in enhancing tumor growth [11, 12]. Introduction of HBx into HCC cell lines can cause cells to enter cell cycle through activation of Src kinase, Ras, and MAPKs [13] or through induction of cyclin expression and cyclin-dependent kinase activity [14]. Inhibition of apoptosis by HBx by elevation of transcription factor nuclear factor Kappa B (NF-*κ*B) has also been linked to the development of HCC [15]. However, the results from several other HBx-transgenic mouse studies do not support the direct link between HBx and tumorigenesis of HCC [16, 17]. In contrast, an inhibitory activity of HBx in hepatoma cell growth has been shown both *in vivo* and *in vitro *[18–21]. Inhibition of proliferation via GSK-3*β*/*β*-Catenin cascade [21], induction of apoptosis via releasing cytochrome c from mitochondria [22], and inactivating FLICE inhibitor protein (c-FLIP) [23] have been proposed for the antigrowth activity of HBx. To develop a complete understanding of HBx-associated liver disease and hepatocarcinogenesis, it will be important to reconcile these apparently conflicting data. 

Besides inflammatory mediators, such as interleukin-6 and interleukin-1, accumulating evidence indicates a critical role of dysregulated growth and survival-related pathways in HCC development [24]. Aberrant activation of Raf-MEK-ERK and PI3K-Akt pathways driven by epidermal growth factor receptor (EGFR) is commonly observed and implicated in the tumor growth and progression of many human cancer types, including HCC [25]. Moreover, activation of EGFR signaling pathways via the overexpression of either its cognate ligands or itself is strongly associated with the poor prognosis of HCC [26, 27]. Interestingly, the poor prognosis is particularly observed in HBV-infected HCC patients with EGFR expression [27, 28]. Activation of oncogenic MAPK and PI3K/Akt signaling pathways is also frequently observed in HBx-expressing HCC cells [29, 30]. These observations imply an association between EGFR and HBx in HBV-associated HCC. However, there is a lack of direct evidence to prove the modulation of EGFR expression by HBx in controlling cellular growth of HCC. 

 In this study, our data surprisingly reveal that HBx decreases, but not increases, cell proliferation of HCC cells by suppressing EGFR protein expression. Mechanically, targeting EGFR mRNA 3′UTR by upregulated microRNA-7 (miR-7) in response to HBx accounts for the suppression of EGFR protein level in HBx-expressing HCC cells. Our data support the inhibitory role of HBx in the cell growth of HBV-associated HCC through the miR-7-depednent EGFR suppression. 

## 2. Materials and Methods

### 2.1. Cell Culture

The human hepatocellular carcinomas Hep3B, HepG2, and their derivatives with HBx expression were cultured in Dulbecco's modified eagle medium: nutrient mixture F-12 (DMEM/F12) supplemented with 10% fetal bovine serum. 

### 2.2. Chemicals, Antibodies, and Reagents

The antibody against EGFR was purchased from Santa Cruz (Santa Cruz, CA), and the antibody against HBx was from Abcam (Cambridge, UK). We purchased antibody against myc-tag from Sigma-Aldrich (St. Louis, MO). The validated siRNAs for negative control, HBx, miR-7 mimic, miR-7 inhibitor, and DharmaFECT 1 transfection reagent were all from Dharmacon (Lafayette, CO). We purchased TransIT-2020 transfection reagent from Mirus Bio LLC (Madison, WI). The QuickGene RNA-cultured cell kit was from Kurabo (Osaka, JP). The RevertAid H Minus First Strand cDNA synthesis kit was purchased from Fermentas (Glen Burnie, MD). The VeriQuest Fast SYBR Green qPCR Master Mix was from Affymetrix (Cleveland, OH). TaqMan Probe qPCR Master Mix was purchased from Roche (Indianapolis, IN).

### 2.3. Transfection and Reporter Gene Assay

The luciferase reporter gene containing full-length 3′UTR of the miR-7-targeting human EGFR gene was a gift from Dr. Keith Giles (Western Australian Institute for Medical Research). Cells with 60–80% of confluence were transfected with 0.5 *μ*g of EGFR-3′UTR luciferase plasmid along with or without different doses of myc-HBx expression vector by using TransIT-2020 transfection reagent according to the manufacturer's instruction. After 48 hrs of transfection, cells lysates were harvested and subjected to luciferase assay system. Luciferase activity was normalized to *β*-gal. For siRNA/microRNA transfection, cells with 60–80% of confluence were transfected with various siRNAs by using DharmaFECT 1 transfection reagent. Cells were harvested at indicated time points and subjected to further experiment. 

### 2.4. Reverse Transcription-Quantitative Polymerase Chain Reaction (RT-qPCR)

Total RNA was extracted by using QuickGene RNA-cultured cell kit according to the manufacture's instruction. One *μ*g of RNA was subjected to reverse transcription with the RevertAid H Minus First Strand cDNA synthesis kit. The qPCR analysis of EGFR and HBx mRNA expressions was performed on ABI 7500 system (Applied Biosystems) by using VeriQuest Fast SYBR Green qPCR Master Mix and was normalized to GAPDH or actin expression. The qPCR analysis of miR-7 expression was performed on LightCycler 480 System (Roche) by using TaqMan Probe qPCR Master Mix and was normalized to the expression of small RNA (U47).

### 2.5. Cell Growth Assay

Cell growth was measured in MTT, cell counting, and crystal violet staining assays. For MTT assay, cells (2–5 × 10^3^ cells/well) were seeded in 96-well plates for indicated time periods, and then 1 *μ*g/mL MTT was added to each well. After 4-hour incubation, formazan was solubilized in 100 *μ*L DMSO/well and the absorbance was measured at 570 nm. For cell counting, cells were trypsinized and relative cell amounts were counted by using Countess Automated Cell Counter (Invitrogen, Carlsbad, CA). For crystal violet staining, cells were seeded with the same amount at the beginning. Five to seven days later, relative cell amounts were determined by crystal violet staining. In brief, cells were washed with 1X PBS once, followed by fixation, and staining with 1% crystal violet in a solvent of 30% ethanol for 15–30 minutes at room temperature. Then, cells were washed with tape water till complete elimination of the background interfered.

## 3. Results

### 3.1. The Protein Level of EGFR Was Attenuated in Response to HBx Expression in HCC Cells

**Figure 1:**
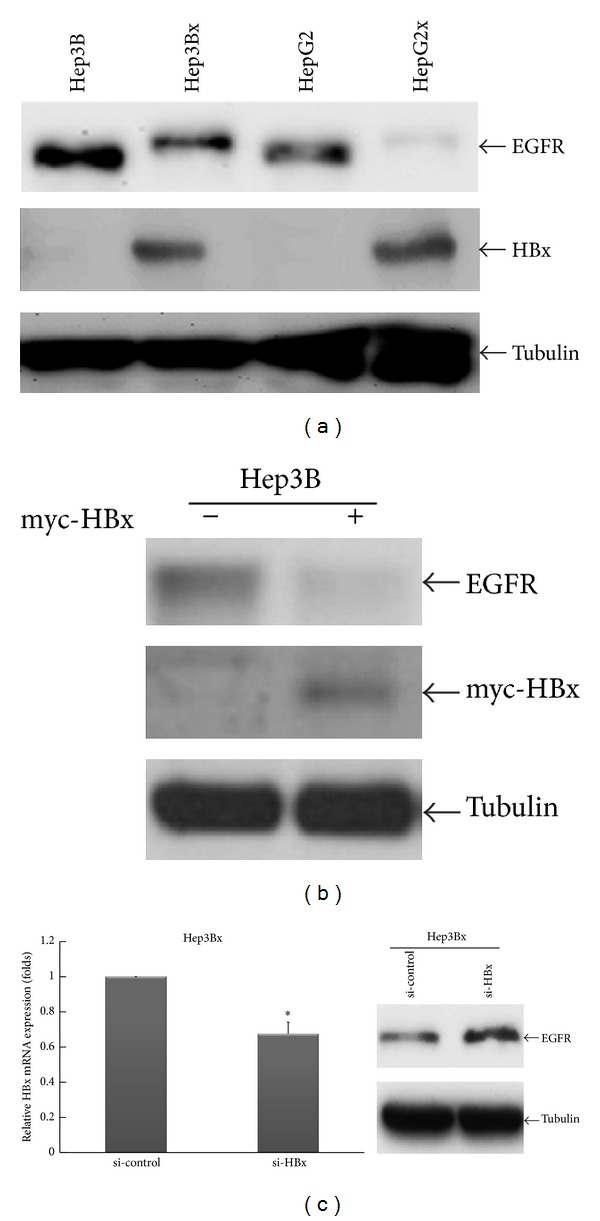
The protein expression of EGFR was attenuated in response to HBx expression in HCC cells. (a) The protein expressions of EGFR, HBx, and Tubulin in HCC cells were analyzed by Western blot. Tubulin acts as an internal control. (b) Hep3B cells were transiently transfected with myc-HBx expression vector for 48 hrs. EGFR protein expression was examined by Western blot. (c) Hep3Bx cells were transiently transfected with si-control or si-HBx for 3 days. The gene silencing for HBx mRNA was determined by RT-qPCR. Under the condition, EGFR protein expression was also analyzed by Western blot. Statistical analysis was performed by Student's *t*-test. *P < 0.05 as compared to the control group.

To investigate the regulatory roles of HBx in EGFR expression, the protein levels of EGFR in Hep3B and HepG2 HCC cell lines and in their HBx-stable transfectants, Hep3Bx and HepG2x cells, were examined by Western blot analysis. Unexpectedly, we found that the protein level of EGFR was obviously reduced in both HBx-expressing Hep3Bx and HepG2x cells as compared with their counterpart Hep3B and HepG2 cells (Figure 1(a)). To rule out the possibility of the EGFR attenuation due to the effects of clonal selection, we transiently enforced HBx expression into Hep3B cells and analyzed EGFR protein expression. As shown in Figure 1(b), the EGFR protein level was decreased by the enforced HBx expression in Hep3B cells. In support to these findings, silencing of HBx with siRNA could restore EGFR protein level in Hep3Bx cells (Figure 1(c)). Taken together, these results indicate an inhibitory effect of HBx on EGFR protein expression in HCC cells.

### 3.2. The 3′UTR Activity of EGFR Was Reduced by HBx in HCC Cells

**Figure 2:**
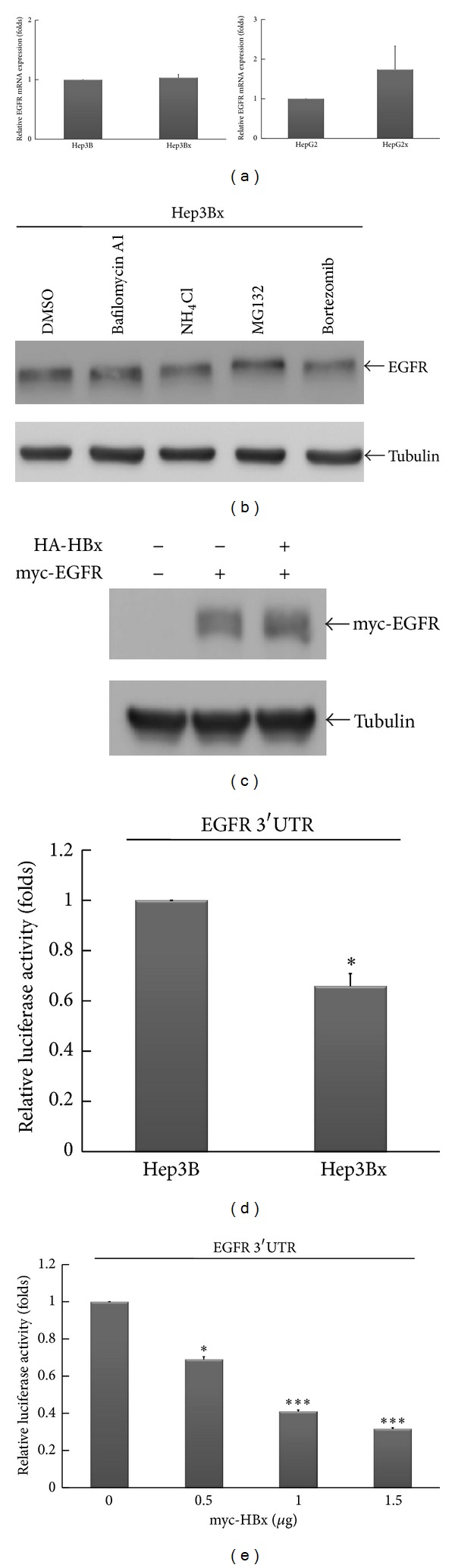
The 3′UTR activity of EGFR was reduced by HBx in HCC cells. (a) The mRNA expression of EGFR in HCC cells was examined by RT-qPCR. The EGFR mRNA expression was normalized to actin expression. (b) Hep3Bx cells were treated with either lysosomal inhibitors (bafilomycin A1 and NH_4_Cl) or proteasomal inhibitors (MG132 and bortezomib) for 6 hrs. EGFR protein expression was analyzed by Western blot. (c) Hep3B cells were transiently transfected with myc-EGFR expression vector along with or without HA-HBx plasmid for 48 hrs. The protein expression of myc-EGFR was examined by Western blot with anti-myc antibody. (d) Hep3B and Hep3Bx cells were transiently transfected with EGFR-3′UTR luciferase plasmid for 48 hrs. Total cells lysates were harvested for luciferase activity analysis. The luciferase activities were normalized to *β*-gal. Values of luciferase activity were means ± SE of three determinations. Statistical analysis was performed by Student's *t*-test. *P < 0.05 as compared to Hep3B cells. (e) Human embryonic kidney HEK293 cells were transiently transfected with EGFR-3′UTR luciferase plasmid as well as different doses of myc-HBx expression vector for 48 hrs. Total lysates were harvested for luciferase activity analysis. The luciferase activities were normalized to *β*-gal. Values of luciferase activity were means ± SE of three determinations. Statistical analysis was performed by Student's *t*-test. *P < 0.05; ***P < 0.001 as compared to control group.

We next addressed the molecular mechanisms of HBx-mediated EGFR suppression. Since the regulations of gene expression by HBx have been widely reported [31–33], we first examined whether HBx reduces EGFR protein expression through transcriptional regulation. However, the mRNA level of EGFR was comparable in Hep3B and Hep3Bx cells (Figure 2(a), left panel) and was even slightly higher in HepG2x cells than in HepG2 cells (Figure 2(a), right panel), suggesting that HBx suppresses EGFR expression through posttranscriptional regulation. It is well documented that EGFR is subjected to polyubiquitination by Cbl and proceeds to endocytosis, followed by lysosomal degradation upon binding with ligands [34, 35]. In addition, the regulation of EGFR activity has been reported to involve proteasomal degradation with unclear molecular mechanisms [36, 37]. We thus examined whether HBx affects EGFR protein expression via these degradation pathways. To this end, both lysosomal and proteasomal inhibitors were applied. As shown in Figure 2(b), however, neither lysosomal inhibitors (bafilomycin A1 and NH_4_Cl) nor proteasomal inhibitors (MG132 and bortezomib) could restore the EGFR protein expression in Hep3Bx cells, suggesting that the HBx-reduced EGFR protein expression is not mediated by enhanced receptor degradation. Moreover, enforced expression of HA-HBx into Hep3B cells did not affect the myc-EGFR protein expression, which is driven by heterologous CMV promoter (Figure 2(c)). These results further indicate that HBx has no effect on both promoter activity and protein stability of EGFR. 

 It is well known that microRNA (miRNA) targets the 3′UTR of mRNA to inhibit protein translation [38]. HBx was recently reported to enhance HCC progression via deregulating miRNA expression [39]. These observations and our results of Figures 2(a)–2(c) led us to further investigate whether HBx affects 3′UTR activity of EGFR mRNA through induction of miRNAs. Accordingly, the luciferase gene constructed with full-length 3′UTR of human EGFR gene was employed. As shown in Figure 2(d), the 3′UTR activity of EGFR in Hep3Bx cells was lower than that in Hep3B cells. Moreover, when the myc-HBx expression was enforced into cells, we observed an attenuation of 3′UTR activity of EGFR by myc-HBx in a dose-dependent manner (Figure 2(e)). Collectively, these results suggest that HBx suppresses EGFR protein expression through targeting its 3′UTR activity.

### 3.3. HBx Upregulated miRNA-7 (miR-7) Expression to Reduce EGFR Protein Level in HCC Cells

**Figure 3:**
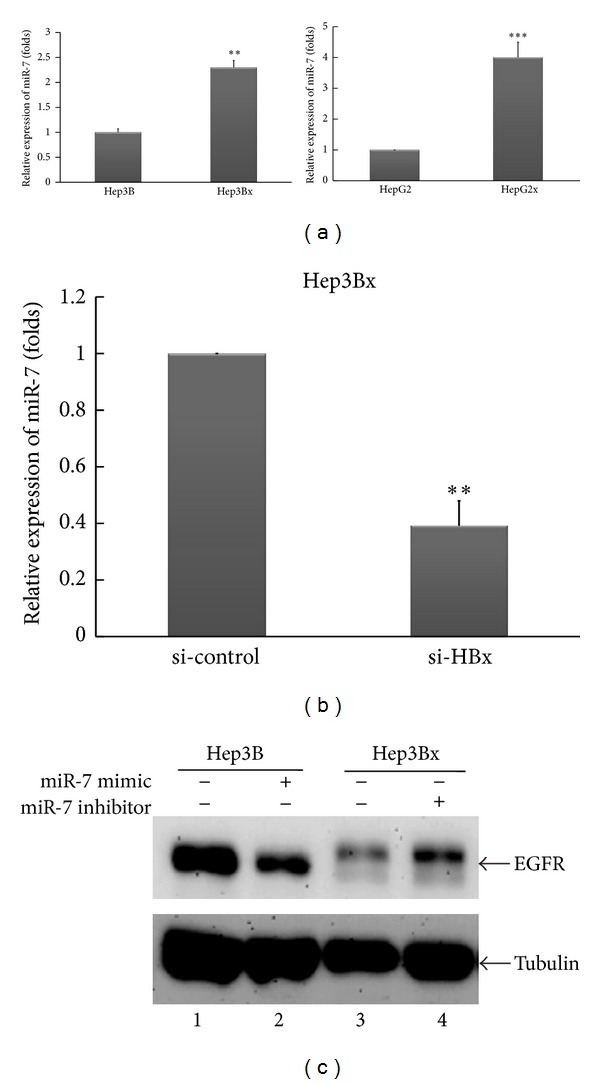
HBx upregulated miR-7 expression to reduce EGFR protein level in HCC cells. (a) The miR-7 expression in HCC cells was examined by RT-qPCR. The miR-7 expression was normalized to small RNA U47 level. Statistical analysis was performed by Student's *t*-test. **P < 0.01; ***P < 0.001 as compared to individual parental cells. (b) Hep3Bx cells were transiently transfected with si-control or si-HBx for 3 days. The miR-7 expression was analyzed by RT-qPCR. The miR-7 expression was normalized to small RNA U47 expression. Statistical analysis was performed by Student's *t*-test. **P < 0.01 as compared to control group. (c) Hep3B and Hep3Bx cells were transiently transfected with miR-7 mimic or miR-7 inhibitor, respectively. Four days later, the EGFR protein expression was analyzed by Western blot.

The mechanism underlying the regulation of EGFR 3′UTR activity by HBx was further explored. It is well documented that miR-7 plays critical roles in the downregulation of EGFR expression in many cancer types [40–43]. The dysregulation of miR-7 leading to HCC progression is also reported by Fang et al., more recently [44]. Thus, we clarified whether HBx upregulates miR-7 expression to target 3′UTR of EGFR mRNA and in turn leads to the attenuation of EGFR protein level. First, we examined the expression of miR-7 in both Hep3B/Hep3Bx and HepG2/HepG2x cell pairs. As shown in Figure 3(a), both Hep3Bx and HepG2x cells presented a higher expression level of miR-7 than their counterparts. To confirm the induction of miR-7 expression by HBx, HBx gene silencing with siRNA was applied. We found that the miR-7 expression in Hep3Bx cells was inhibited by HBx siRNA (Figure 3(b)), supporting that HBx suppresses EGFR protein level through inducing miR-7. That is, adjustment of miR-7 expression could modulate the protein level of EGFR presented in HCC cells. Indeed, when miR-7 expression was enforced into Hep3B cells, EGFR protein level was decreased (Figure 3(c), compared lane 2 with lane 1). On the contrary, when miR-7 expression was deprived from Hep3Bx cells by using miR-7 inhibitor, the EGFR protein expression was increased (Figure 3(c), compared lane 4 with lane 3). Altogether, these results indicate that HBx upregulates miR-7 expression to downregulate the protein level of EGFR.

### 3.4. The miR-7-Dependent EGFR Suppression by HBx Slows Down Cell Growth in HCC

**Figure 4:**
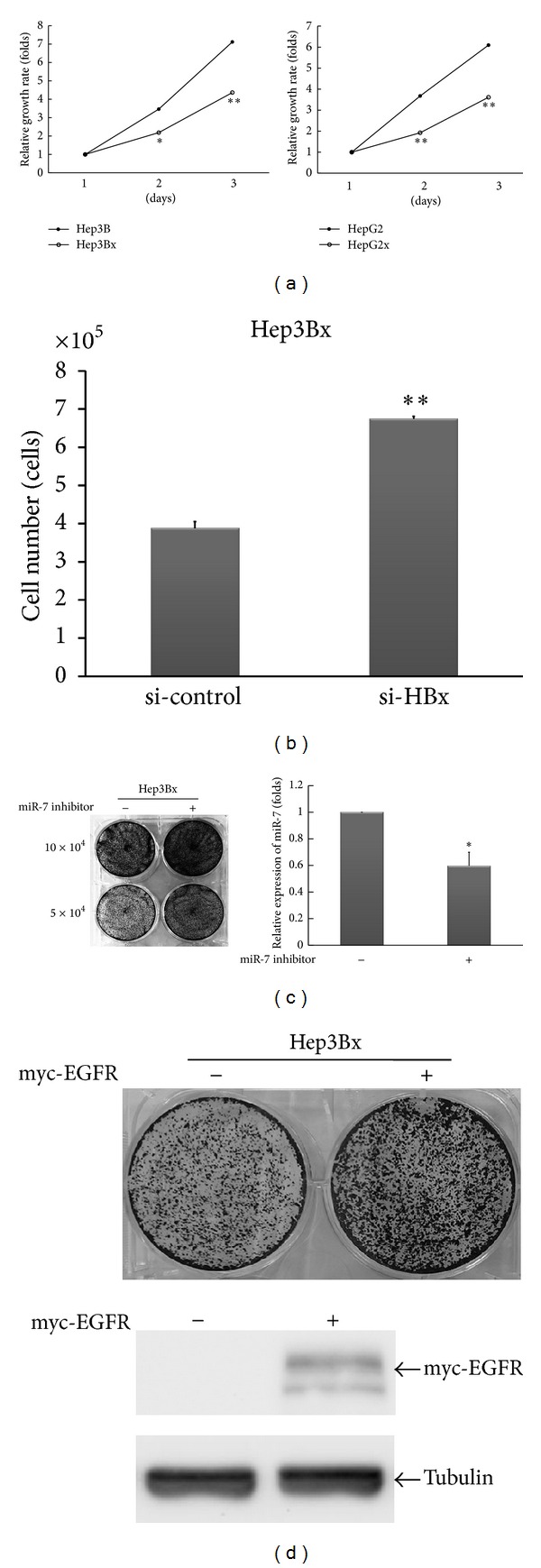
The regulatory trait of HBx-miR-7-EGFR conferred HCC cells a slow growth behavior. (a) The growth curves of Hep3B, HepG2, and their derivatives were determined by MTT assay. Statistical analysis was performed by Student's *t*-test. *P < 0.05; **P < 0.01 as compared to individual parental cells. (b) Hep3Bx cells were transiently transfected with si-control and si-HBx for 4 days. These cells were then trypsinized for cell number counting. Statistical analysis was performed by Student's *t*-test. **P < 0.01 as compared to control group. (c) Hep3Bx cells were transiently transfected with or without miR-7 inhibitor for 1 day. Cells were reseeded at the same amount between groups and allowed for growth. The growth rate was determined by crystal violet staining. Total RNA was also collected for examination of miR-7 expression by RT-qPCR. (d) Hep3Bx cells were transiently transfected with or without myc-EGFR expression vector for 1 day. Similar procedures as described in (c) were performed. The protein expression of myc-EGFR was examined by Western blot.

Our above results led us to further investigate the impact of HBx-mediated EGFR suppression on HCC. It is known that EGFR signaling is a strong mitogenic stimulator for cell proliferation, and a slow cell growth is expectable when EGFR expression is reduced. Therefore, we examined the growth curve in both HCC cells lines and their HBx-expressing derivatives. As expected, HCC cells with HBx expression, including Hep3Bx and HepG2x cells, showed a retarded growth rate as compared with their counterparts (Figure 4(a)). Consistently, HBx-expressing Hep3B cells exhibited a delayed cell cycle as evidenced by the increased cell accumulation in G0/G1 phase (Figure S1(a), see in Supplementary Material available online at http://dx.doi.org/10.1155/2013/682380) and longer duration of S phase (Supplementary Figure S1(b)) when compared with their counterparts. Moreover, the cell number was increased in Hep3Bx cells after HBx expression was silenced by siRNA for 4 days (Figure 4(b)). These results suggest that HBx may slow down HCC proliferation through downregulation of EGFR expression in a miR-7-dependent manner. Indeed, introduction of miR-7 inhibitor (Figure 4(c)) or myc-EGFR (Figure 4(d)) into Hep3Bx cells could significantly increase the cell growth as determined by crystal violet staining. Collectively, these results indicate that the miR-7-dependent EGFR suppression by HBx reduces cell growth of HCC.

## 4. Discussion

 In this study, our data showed downregulation of EGFR protein level by HBx in HCC cells (Figure 1). As a consequence, it rendered HCC cells with HBx expression to display a phenotype of slow growth (Figure 4), which is consistent with the previous findings that HBx plays an inhibitory role in the HCC cell growth both *in vivo* and *in vitro* [18, 20, 21, 45–47]. In contrast, HBx has been proposed to positively regulate cell proliferation and metastatic ability of HCC tumor cells [48]. There also has been considerable confusion regarding both proapoptotic and antiapoptotic functions of HBx mediated by p53-dependent and -independent manners during hepatocarcinogenesis [49, 50]. The differences in cell contexts and experimental condition used in a particular system may explain these conflict observations [5]. 

 Of note, carboxy-terminal (C-terminal) truncation of HBx is frequently observed in HCC patients with HBV infection [51, 52]. It has further been observed that overexpression of C-terminal truncated HBx leads to cell growth of HCC [19, 46], suggesting an inhibitory role of carboxy-terminal domain of HBx in controlling cell proliferation. Consistently, overexpression of centromere protein A (CENP-A), a protein required for chromosome segregation in mitosis, has been found to be closely associated with HBx carboxy-terminal mutation in HCC [53]. The enhancement of proliferation and cyclin D1 expression by HBx carboxy-terminal deletion mutant (deleted at nucleotide 382–400) in LO2 hepatocyte cells further supports the inhibitory role of C-terminal domain of HBx in controlling cell proliferation [54]. The HBx used in this study is full-length and does not contain this deletion, raising the possibility that C-terminal domain of HBx may be responsible for HBx-mediated EGFR suppression. Since it has been shown that HBx-mediated regulation of NF-*κ*B activity varies depending on the residues of HBx point mutations [55], it is also worthy to explore in the further studies whether any point mutation in HBx determines its ability to suppress EGFR expression.

 Dysregulation of miRNA expression has been widely observed in HCC [56, 57]. Wang et al. first demonstrate that HBx can regulate miRNA expression [39]. Several studies also explore the pathological functions of aberrant miRNA expression in HCC in response to HBx [29, 58–60]. Our data further revealed that the molecular mechanism underlying HBx-mediated EGFR suppression is due to the induction of miR-7, which can bind to and target EGFR 3′UTR, leading to the downregulation of EGFR protein level (Figures 2 and 3). Disruption of the miR-7-EGFR regulatory trait increases the growth rate of HBx-expressing HCC cells, suggesting that HBx induces miR-7 to reduce EGFR expression and cell growth (Figure 4). In consistence with our findings, dysregulated miR-7 is recently detected in tumor tissues from HCC patients and functions in suppressing cell growth by targeting Akt/mTOR, a survival signaling pathway downstream of EGFR [44]. Indeed, overexpression of miR-7 resulted in the attenuation of Akt activity (Supplementary Figure S2) and it is frequently reported to have an inhibitory effect on tumor growth in various cancer types [44, 61–63], supporting the tumor suppressive roles of miR-7. 

 Although the induction of miR-7 by HBx was demonstrated for the first time in this study, further investigations remain to understand the underlying regulatory mechanism. In addition to being targeted by miR-7, EGFR has also been reported to induce miR-7 transcription relying on its tyrosine kinas activity [64], suggesting miR-7 as a negative feedback regulator of EGFR expression. However, our data showed that miR-7 is constitutively increased in stable HBx-expressing cells even if EGFR expression is attenuated, indicating that other mechanisms rather than EGFR signaling mediate HBx-induced miR-7 expression. Interestingly, induction of miR-7 is selectively found in differentiating neuronal progenitor cells with overexpression of IKK*α*, an upstream kinase for activation of NF-*κ*B [65]. HBx has been widely found to interact with NF-*κ*B to regulate gene expressions involved in the HCC pathogenesis [31–33]. Our previous findings also showed that IKK*α* is activated by HBx and translocates into the nucleus to function as a chromatin modifier for gene transcription. These observations raise the possibility that HBx may induce miR-7 expression through IKK/NF-*κ*B and nuclear IKK*α* signaling pathways in HCC cells, which deserves further investigations. 

## 5. Conclusion

 This study linking viral regulatory protein HBx to EGFR suppression reveals an inhibitory role of HBx in the cell growth of HCC. HBx increases the expression of miR-7 and subsequently leads to the attenuation of EGFR protein expression, which reflects a slow-growth phenotype of HBx-expressing HCC cells. Our findings not only identify that miR-7 is a novel regulatory target of HBx, but also enhance the understandings of the pleiotropic roles of HBx in HBV-associated HCC.

## Supplementary Material

HBx expression slowed down the growth rate of Hep3B cells (Figure 4(a)). Consistently, HBx- expressing Hep3B cells also exhibited a delayed cell cycle as evidenced by the increased cell accumulation in G0/G1 phase (Supplementary Figure S1(a)) and longer duration of S phase (Supplementary Figure S1(b)) when compared with their counterparts. “This effect may be resulted from the downregulation of EGFR signaling by HBx-elevated miR-7 expression. Indeed, overexpression of miR-7 also resulted in the attenuation of EGFR downstream Akt activity (Supplementary Figure S2).

## Abbreviations

HBV: Hepatitis B virus
HCC: Hepatocellular carcinoma
HBx: HBV-encoded X protein
EGFR: Epidermal growth factor receptor
miR: MicroRNA
3′UTR: 3′untranslated region
HCV: Hepatitis C virus
ORF: Open reading frame
NF-*κ*B: Nuclear factor Kappa B
c-FLIP: FLICE inhibitor protein
C-terminal: Carboxy-terminal
CENP-A: Centromere protein A.
