# Supplementary material for: Hepatitis B Virus-Encoded X Protein Downregulates EGFR Expression via Inducing MicroRNA-7 in Hepatocellular Carcinoma Cells
Source: Evid Based Complement Alternat Med. 2013 Jun 11;2013:682380. doi: 10.1155/2013/682380 (PMC3693120; doi:10.1155/2013/682380)
Supplement: Supplementary file 1 — HBx expression slowed down the growth rate of Hep3B cells (Figure 4(a)). Consistently, HBx- expressing Hep3B cells also exhibited a delayed cell cycle as evidenced by the increased cell accumulation in G0/G1 phase (Supplementary Figure S1(a)) and longer duration of S phase (Supplementary Figure S1(b)) when compared with their counterparts. “This effect may be resulted from the downregulation of EGFR signaling by HBx-elevated miR-7 expression. Indeed, overexpression of miR-7 also resulted in the attenuation of EGFR downstream Akt activity (Supplementary Figure S2). [file 682380.f1.pdf]

# Supplementary Information

## SUPPLEMENTARY FIGURE S1

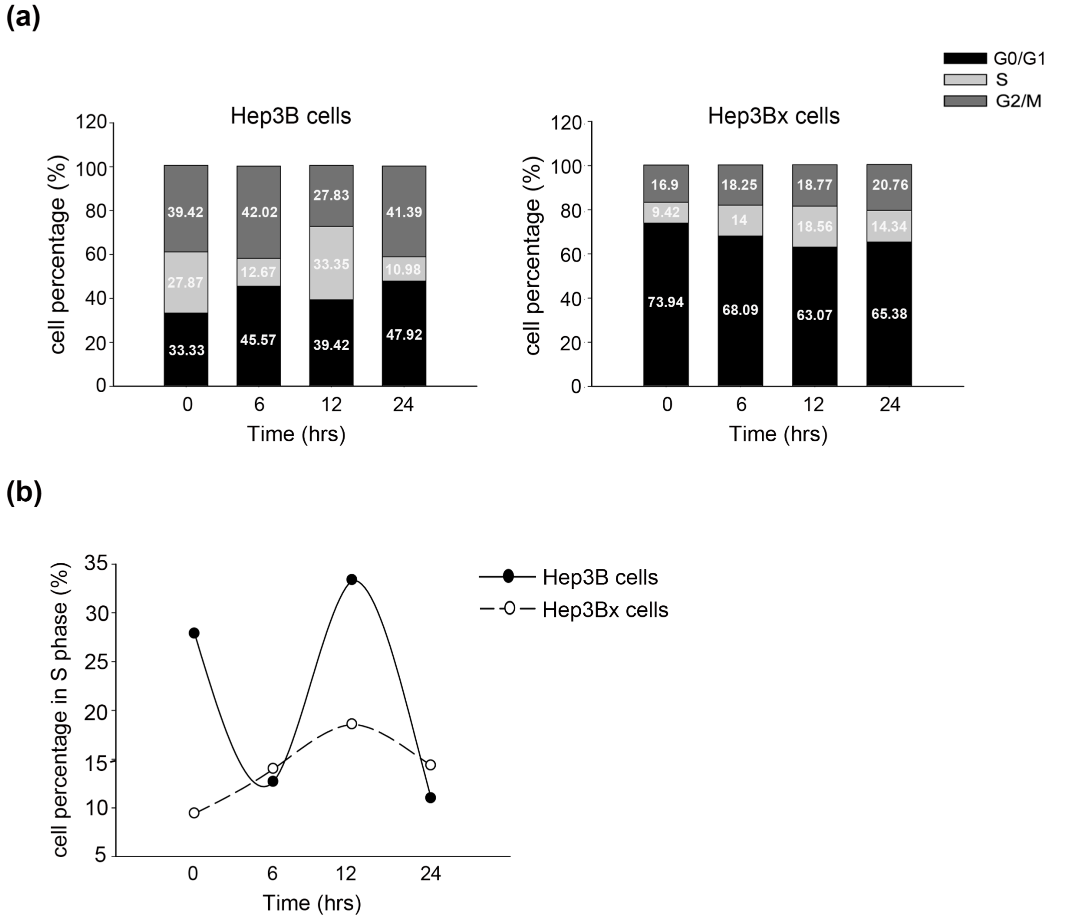


**SUPPLEMENTARY FIGURE S1: Hep3Bx cells displayed a delayed cell cycle.** (a-b) After Hep3B and Hep3Bx cells were seeded overnight, both cell lines were fixed at different time points and subjected to flow cytometry analysis. The cell cycle distribution of both cell lines was analyzed and quantified (a). The percentage of population in S phase at different time periods was shown in (b).

## SUPPLEMENTARY FIGURE S2

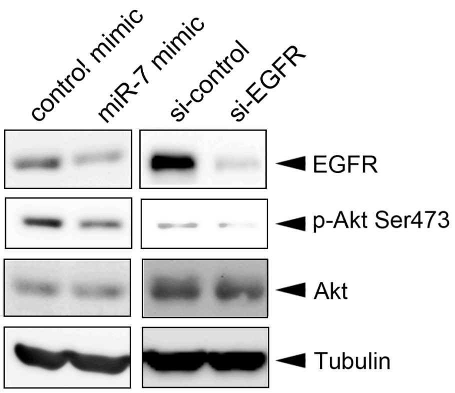


**Supplementary Figure S2: Overexpression of miR-7 or deprivation of EGFR expression attenuated the phosphorylation level of Akt in Hep3B cells.** Hep3B cells were transiently transfected with either miR-7 mimic or EGFR siRNA. Three days later, whole cell lysates were harvested and subjected to western blot analysis. Various protein expressions were examined by indicated antibody. Akt activation was assessed by p-Akt Ser473 antibody.
